# Nurses’ Perceptions of a Care Plan Information Technology Solution With Hundreds of Clinical Practice Guidelines in Adult Intensive Care Units: Survey Study

**DOI:** 10.2196/11846

**Published:** 2019-02-12

**Authors:** Azizeh Khaled Sowan, Meghan Leibas, Albert Tarriela, Charles Reed

**Affiliations:** 1 School of Nursing University of Texas Health at San Antonio San Antonio, TX United States; 2 Center for Clinical Excellence University Health System San Antonio, TX United States; 3 Transplant Cardiac Intensive Care Unit University Health System San Antonio, TX United States

**Keywords:** usability, patient care planning, evidence-based practice, nursing, documentation, information technology, survey

## Abstract

**Background:**

The integration of clinical practice guidelines (CPGs) into the nursing care plan and documentation systems aims to translate evidence into practice, improve safety and quality of care, and standardize care processes.

**Objective:**

This study aimed to evaluate nurses’ perceptions of the usability of a nursing care plan solution that includes 234 CPGs.

**Methods:**

A total of 100 nurses from 4 adult intensive care units (ICUs) responded to a survey measuring nurses’ perceptions of system usability. The survey included 37 rated items and 3 open-ended questions.

**Results:**

Nurses’ perceptions were favorable with more than 60.0% (60/100) in agreement on 12 features of the system and negative to moderate with 20.0% (20/100), to 59.0% (59/100) in agreement on 19 features. The majority of the nurses (80/100, 80.0% to 90/100, 90.0%) agreed on 4 missing safety features within the system. More than half of the nurses believed they would benefit from refresher classes on system use. Overall satisfaction with the system was just above average (54/100, 54.0%). Common positive themes from the narrative data were related to the system serving as a reminder for complete documentation and individualizing patient care. Common negative aspects were related to duplicate charting, difficulty locating CPGs, missing unit-specific CPGs, irrelevancy of information, and lack of perceived system value on patient outcomes. No relationship was found between years of system use or ICU experience and satisfaction with the system (*P*=.10 to *P*=.25).

**Conclusions:**

Care plan systems in ICUs should be easy to navigate; support efficient documentation; present relevant, unit-specific, and easy-to-find information; endorse interdisciplinary communication; and improve safety and quality of care.

## Introduction

### Background

Usability of health information technology (IT) is essential yet an overlooked aspect that drives system fitness to care context, adoption, and quality and safety of care [1-5]. Usability is “the effectiveness, efficiency, and satisfaction with which specific users can achieve a specific set of tasks using a specific system in a particular environment” [6]. Methods for usability evaluation of health IT include questionnaires, chart review, log file analysis, and observation of user-system-task- environment interaction [7]. Questionnaires are commonly used in usability studies to understand end-user perception of IT, are easy to administer, and serve as a basis for subsequent rigorous usability testing using techniques such as user-system- task-environment interaction. In this study, we assessed nurses’ perceptions of a care plan IT solution within the nursing documentation system in intensive care units (ICUs). The solution allows nurses to integrate the recommendations from hundreds of clinical practice guidelines (CPGs) into the plan of care.

CPGs are documents that synthesize recent research findings and recommend a plan of care to diagnose, treat, and manage disease conditions and symptoms. CPGs are essential treatment components for standardized evidence-based practice (EBP), better patient outcomes, cost reduction, and compliance with national safety standards [8-13]. On the other hand, CPGs are lengthy complex documents and vary in their trustworthiness, specificity, strength of evidence, and clarity of recommendations, thus hindering their adaption in intensive care environments with urgent and complex medical conditions [14-16]. Promising strategies for implementation and adoption of CPGs have focused on automating essential components (ie, the recommendations) of the CPGs and integrating them into the electronic health record (EHR) using interactive clinical decision support systems in the forms of alerts and reminders, care protocols, and bundles [12-17]. Although these approaches were successful in some contexts, they allow automating a limited number of CPGs and in many cases produce a small adoption and adherence rate in addition to alert fatigue [15-18]. Although the integration of CPGs’ recommendations into an EHR is complex and multifaceted, in many cases and based on end users’ perspectives, poor adherence to automated CPGs is attributed to poor usability of the IT system [17-21].

To improve adoption of CPGs, Elsevier Clinical Practice Model Resource Center developed *Care Planning*, a comprehensive interdisciplinary care plan and documentation solution that provides clinicians instant point-of-care access to recommendations from hundreds of CPGs for assessment, diagnosis, treatment, and evaluation [22]. Care Planning is developed based on the Elsevier Clinical Practice Model Framework. The framework places the patient as the center of care and focuses on the core beliefs, principles, and theories of EBP, health and healing, interdisciplinary integration, partnership, health informatics, and international consortium. The Care Planning CPGs, which were developed by interdisciplinary clinicians, are updated periodically and are tested by the Elsevier Clinical Practice Consortium that includes more than 400 hospitals [22]. Care Planning is currently used by many health care institutions across the United States and Canada [22]. The integration of an IT solution such as Care Planning into the nursing documentation system in complex environments such as ICUs is likely to have mixed effects on care processes and quality and safety outcomes. Despite the rise in system adoption, little information is available about the value and usability of the system from a nursing perspective.

### Objective

In our facility, Care Planning is known as Knowledge-Based Charting (KBC) and is a major part of the nursing documentation system used to plan and document standardized and evidence-based nursing care. This study describes nurses’ perceptions of the usability of the KBC solution within the nursing documentation system in terms of ease of use and documentation, usefulness, efficiency, system safety features, help resources, and training on system use.

## Methods

### Design, Setting, and Sample

This descriptive study took place in 4 adult ICUs in a 705-bed university teaching hospital with a large referral base in the southwest of the United States. ICUs included neuro (NeuroICU), medical (MICU), surgical trauma (STICU), and transplant and cardiac (TCICU), and had a total of 206 nurses and 950 annual discharges and transfers. After obtaining the approval of the institutional review board, 100 nurses were invited to respond to a questionnaire measuring their perceptions of the usability of the KBC solution. Recruitment was stopped after the target sample of 100 nurses was reached.

### Description of the Knowledge-Based Charting System

In our facility, KBC (release 3.2) was integrated into the nursing documentation system in the EHR (Sunrise, Allscript). KBC consists of the index and flowsheets of CPGs. The CPGs’ index is a database that includes 165 medical and surgical CPGs (eg, acute coronary syndrome and postoperative) and 69 behavioral or human response CPGs (eg, pain and anxiety). The CPGs’ flowsheets are seamlessly integrated into the nursing documentation system only when a CPG is selected from the CPGs’ index as described below. Nurse unit educators and superusers support individual training needs on KBC use. All ICUs were sufficiently equipped with hardware for EHR use.

**Figure 1:**
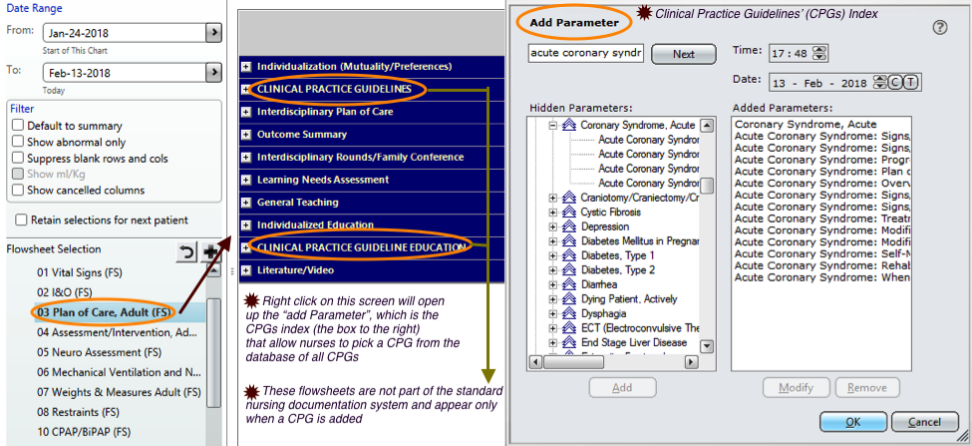
Accessing the clinical practice guideline (CPG) index and adding CPGs from the CPGs’ index.

**Figure 2:**
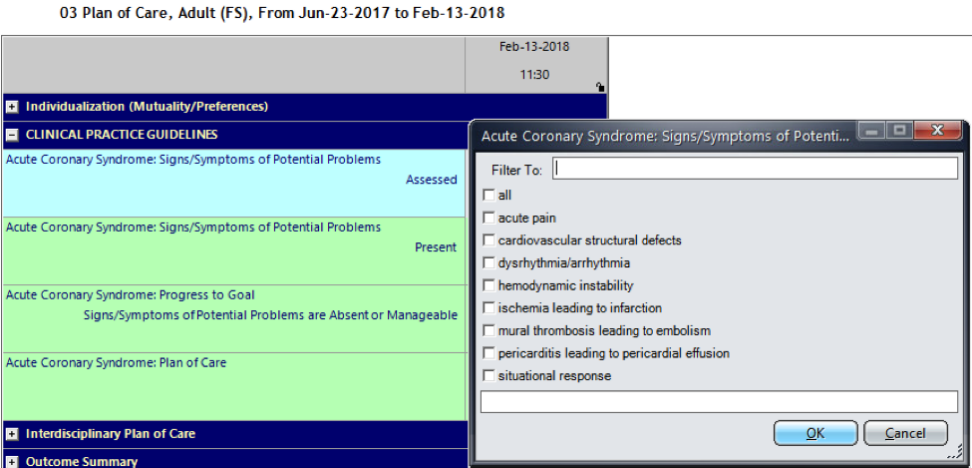
Screens under the clinical practice guidelines’ flowsheet with a side box for “problems assessed”.

The EHR provided nurses complete access to patient information. One of the main fields used by nurses in Sunrise is the plan of care (left-side list, Figure 1). Nurses can add a CPG by clicking on the list that appears under the plan of care (Figure 1). This allows nurses to access the CPGs index (Figure 1 —“Add Parameter”). From this index, nurses select CPGs that are pertinent to the patient condition. Once added, CPG recommendations appear as two main flowsheets under the plan of care list: CPGs flowsheet and CPGs education (Figure 1). Each of these flowsheets has subscreens to be completed by nurses once clicked. For example, the CPGs flowsheet has the following 4 subscreens (Figure 2): signs and symptoms of potential problems assessed, signs and symptoms of potential problems present, progress to goal, and plan of care. When nurses click any of these subscreens, a side list is presented for nurses to select what they assessed (problems assessed); what exists (problems present); if the goal to progress is improving, declining, or had no changes (progress to goal); and if the interventions related to present problems are ongoing or need to be discontinued or changed (plan of care; see Figure 2).

**Figure 3:**
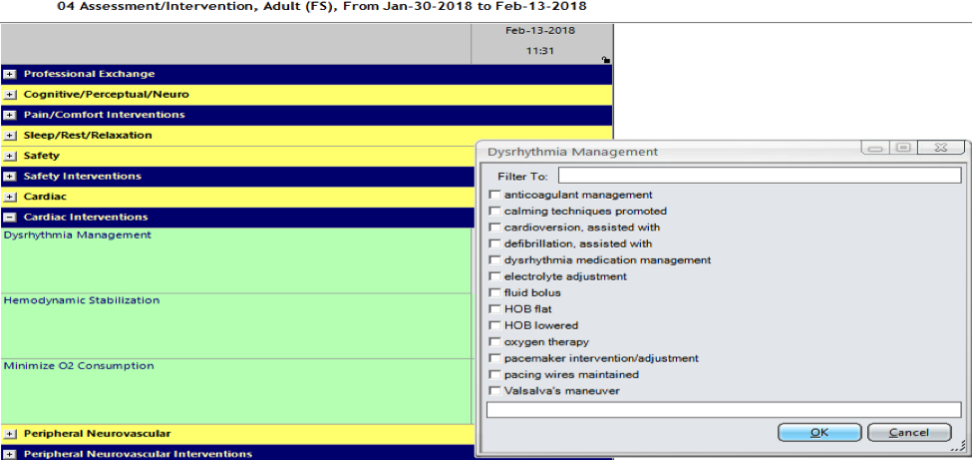
Dysrhythmia management interventions suggested by the dysrhythmia clinical practice guideline.

In addition to the CPG-related flowsheets integrated under the plan of care in Sunrise, 2 other flowsheets are also automatically added to the assessment and intervention field in the nursing documentation system in Sunrise (Figure 3) once a CPG is added to the nursing documentation system. The tabs provide nurses with lists of assessment points and interventions to pick from (see Figure 3, a side screen for dysrhythmia management interventions). As the lists can be lengthy, nurses were trained to choose wisely from these lists to provide manageable care, especially when multiple CPGs apply to the patient condition. Under the assessment and intervention field, standard of care–related fields are highlighted in a different color (yellow and blue) than the CPG-related flowsheets (green).

After a hand-off report, nurses can verify, modify, add, or discontinue CPGs based on the chief medical diagnosis, physical examination, assessment findings, problems list, vital signs, and intake and output. Nurses may also access the complete document of any CPG to confirm the appropriateness of the selected CPG or to learn more about the health condition.

The complete document of a CPG is similar to a CPG summary published by the Guideline Central [23] in which it provides a summary of the recommendations based on the latest evidence for assessment and interventions along with the strength of the recommendation. However, unlike the CPG summary from the Guideline Central, our complete document in the KBC was limited to 3 of the following main parts: the name of the CPG and the target population (ie, asthma, adult patients); goals and outcomes; and assessment, intervention, clinical reasoning, and decision making. The 2 components under goals and outcomes are (1) signs and symptoms of manageable potential problems (ie, hypoxia, pneumonia, and depression) and (2) educational outcomes (ie, symptoms of asthma, medication treatment plan, modifiable risk factors, and self-management strategies). The assessment, intervention, clinical reasoning, and decision- making section of a complete CPG document in KBC includes the following information under each potential problem: definition; assessment strategies; recommended interventions; citation of the evidence; and the strength of the recommendation for each intervention.

### Instrumentation and Procedures for Data Collection

Nurse Perception of the Usability of the KBC Solution Questionnaire was developed after extensive review of usability literature and IT issues identified in critical care settings [1-7,24-33] and was guided by the Davis Technology Acceptance Model [34], Nielsen usability heuristics [35], and Zhang and Walji usability principles [36]. The questionnaire includes 3 sections: (1) demographic data (eg, age, gender, employment status, and years of experience); (2) 37 rated items of a 5-point Likert-type scale of agreement; (3) and 3 open-ended questions to understand missing CPGs that nurses wish the system included and advantages and negative aspects of the KBC. Rated items reflect the following usability aspects of the KBC: perceived ease of use, usefulness (ie, KBC effect on workflow, safety, quality of care, communication, and supporting interdisciplinary care), inclusiveness of the KBC to most important CPGs necessary for ICU conditions, system safety, efficiency, adequacy of training and help resources, and nurse satisfaction with the KBC system. The questionnaire was validated by 5 expert ICU nurse educators, 3 KBC superusers, and informatics experts for appropriateness and adequacy of items and was administered via SurveyMonkey.

To improve the response rate, 2 stations with 4 computers each were set outside the ICUs and nurses were invited to respond to the survey when they were coming to their shifts or leaving the unit. Data collectors also rounded in ICUs before and after shift change to encourage participation. A small cash token for participation was given to each respondent. To foster voluntary participation, some data collectors were non-ICU nurses and data collectors who were ICU nurses administered the questionnaire in ICUs other than their units.

### Analysis

Demographics and survey items were presented using descriptive statistics. The relationship between demographic variables and survey items were examined using correlation tests such as chi-square and Spearman rho, with a significance level of .05. Content analysis was used to categorize narrative data into themes.

## Results

### Nurse Characteristics

All nurses provided complete questionnaires (N=100). As shown in Table 1, the majority of the nurses were from STICU (28/100, 28.0%), aged more than 30 years (60/100, 60.0%), females (65/100, 65.0%), and full-time employees (69/100, 69.0%). Most of the nurses worked in ICUs for 3 years or fewer (59/100, 59.0%) and rated their computer skills as moderate or above moderate (85/100, 85.0%). Of the nurses, 27.0% (27/100) had less than 1-year experience of KBC system use.

**Table 1:** Nurse characteristics (N=100).

Characteristic		n (%)
**Intensive care unit**		
	Surgical trauma	28 (28.0)
	Transplant/cardiac	26 (26.0)
	Medical	26 (26.0)
	Neuro	20 (20.0)
**Age (years)**		
	Less than 30	40 (40.0)
	More than 30	60 (60.0)
**Gender**		
	Female	65 (65.0)
	Male	35 (35.0)
**Employment status**		
	Full-time	69 (69.0)
	Part-time	31 (31.0)
**Experience with KBC^a^ system**		
	Fewer than 6 months	14 (14.0)
	6-11 months	13 (13.0)
	1-3 years	38 (38.0)
	More than 3 years	35 (35.0)
**Years in intensive care units**		
	Fewer than or equal to 3 years	59 (59.0)
	More than 3 years	41 (41.0)
**Years working as a nurse**		
	Fewer than or equal to 3 years	33 (33.0)
	More than 3 years	67 (67.0)
**Level of computer expertise**		
	Novice	0 (0.0)
	Moderate	37 (37.0)
	Above moderate	48 (48.0)
	Expert	15 (15.0)

^a^KBC: Knowledge-Based Charting.

### Nurse Perception of the Usability of the Knowledge-Based Charting System

The internal consistency reliability of the questionnaire was acceptable (Cronbach alpha=.82). Nurses’ responses to the rated survey items reflecting their perceptions of the usability of the KBC system were coded as *Agree* for agree or strongly agree responses and *Disagree* for disagree or strongly disagree responses. Items with a neutral response remained *Neutral* (see Multimedia Appendix 1).

The majority of the nurses agreed that there is a need for the system to suggest most critical interventions, alert nurses for safety considerations and when inappropriate CPGs were selected, and to provide a summary of changes in the patient care plan (Items 1, 2, 4, and 5; 80/100, 80.0% to 90/100, 90.0%).

Although the system includes CPGs for the majority of the medical conditions (Item 6; 74/100, 74.0% agreement), it is missing important CPGs for medical conditions often seen in ICUs (Item 12; 63/100, 63.0% agreement). The majority of the nurses (63/100, 63.0% to 72/100, 72.0%) believed the system helps them with medical conditions that they were not familiar with (Item 7), promotes patient engagement (Item 13), individualizes patient care (Item 14), improves the quality of nursing documentation (Item 15), and provides comprehensive nursing care (Item 16). On the contrary, only one half of the nurses agreed that they see the value of nursing documentation on patient outcomes using the system (Item 24; 54/100, 54.0%) and that the system has positive effects on patient outcomes (Item 29; 49/100, 49.0%).

The majority of the nurses agreed to the consistency of terminologies used to display CPGs and CPGs’ components (Item 11; 64/100, 64.0% and Item 8; 70/100, 70.0%) and more than half agreed to the ease of use of some features of the system (Items 3, 10, 19, 25, and 28; 52/100, 52.0% to 84/100, 84.0%). Yet, only 37.0% (37/100) to 48.0% (48/100) of the nurses believed it is easy to locate CPGs, the documentation of the multidisciplinary team, and the nursing documentation by other disciplines (Items 30, 31, 32, and 33). In addition, although 59.0% (59/100) to 69.0% (69/100) of the nurses considered themselves proficient system users (Item 9) and reported availability of help resources for system use (Item 17), only 58.0% (58/100) reported receiving adequate training on system use (Item 20), and 56.0% (56/100) believed they would benefit from refresher training classes (Item 22).

According to the nurses, the KBC system did not improve documentation efficiency (Item 35; 45/100, 45.0%) and only one-fifth of the nurses believed that the quality of their work is based on the KBC system (Item 37; 20/100, 20.0%). The majority of the nurses (59/100, 59.0%) used workarounds when interacting with the system (Item 18). The overall nurse satisfaction with the system was just above average (Item 26; 54/100, 54.0%).

### Open-Ended Questions

#### Missing Clinical Practice Guidelines

A total of 76 nurses (NeuroICU [17/20], STICU [15/28], MICU [23/26], and TCICU [21/26]) listed missing CPGs that nurses would have liked the system to include. Nurses from the NeuroICU suggested 15 CPGs (eg, mechanical ventilation, neurological diseases, multiple sclerosis, and embolic stroke). STICU nurses suggested 11 CPGs (eg, snakebites, hemodynamics, more trauma-related CPGs, postoperative, and gastrointestinal), whereas MICU nurses suggested 30 (eg, gastrointestinal bleeding, acute liver/renal failure, pulmonary embolism, and respiratory/congestive heart failure). Nurses from TCICU listed the following CPGs as missing: liver and lung transplant, trauma, coronary artery bypass grafting, and toxic ingestion of a specific drug. Gastrointestinal bleeding, flaps, altered mental status, and toxic ingestion of a specific drug were reported by nurses from 2 or more ICUs.

#### Advantages of the System

A total of 88 nurses (NeuroICU [15/20], STICU [26/28], MICU [23/26], and TCICU [24/26]) listed advantages of the KBC system. Major themes with examples are presented in Multimedia Appendix 2. The 2 most common themes were related to the system (1) serving as a reminder to provide complete care and (2) helping nurses organize patient care and track progress toward achieving individualized patient outcomes. The least commonly reported themes were related to ease of system use and system role in promoting accountability and EBP and educating nurses on new medical conditions, specifically the new hires.

#### Negative Aspects of the Knowledge-Based Charting System

A total of 90 nurses (NeuroICU [18/20], STICU [25/28], MICU [23/26], and TCICU [24/26]) provided details on difficulties and negative aspects of using the KBC. Common themes with examples are presented in Multimedia Appendix 3. Duplicate charting and time consuming were the most commonly cited negative aspects of the system. A total of 20 nurses related this to repetitive interventions as a result of lack of communication across CPGs, especially when a patient has multiple CPGs, and lack of cross-communication among different flowsheets; this required the nurses to spend a long time documenting care and negatively affected the time spent with patients.

Difficulty finding appropriate or specific CPGs was another negative aspect contributing to a long time of system use. Nurses suggested listing CPGs by body systems (eg, “if it was listed by systems it would be easier to find”), the use of search features or a search engine instead of viewing a long list of CPGs, and the need for the system to automatically suggest and display related CPGs based on the medical diagnosis. In addition, some of the CPGs are too broad and others are missing from the system.

A total of 25 nurses reported that the system has no value to patient care or nursing and that they select CPGs and complete the documentation for legal purposes only. A careful examination of the data showed that all these nurses have more than 3 years of experience in system use. The complexity of the system also resulted in selecting CPGs at the end of the shift for documentation purposes only instead of using CPGs at the beginning of the shift to guide care. The least common themes were related to system lack-of-safety features and lack of training on system use, specifically for the new hires.

#### Relationship Between Variables

No significant correlations were found between years of experience in KBC system use, ICU, age, years in ICU, and satisfaction with the system (*P*=.10 to *P*=.25).

## Discussion

### Principal Findings

To the best of our knowledge, this is the first study to assess nurses’ perceptions of the usability of a care plan and documentation system that is based on hundreds of CPGs in ICUs. Nurses’ perceptions were favorable on 12 out of 37 features of the system, with more than 60.0% agreement. These were related to ease of use of some features (eg, add and discontinue CPGs) and system usefulness to nursing and patient care (eg, educates nurses on medical conditions and engages the patient in care). On the other hand, nurses reported moderate perceptions with 50.0% to 59.0% agreement on 10 features of the system (eg, training on system use, ease of use and navigation, relevancy of information to nursing care, system support to nursing workflow and information need, and perceived value of nursing documentation on patient outcomes). Negative perceptions were reported on 9 features of the system (20.0% to 49.0% agreement) related to system effect on patient outcomes, difficulty in locating CPGs, lack of system support to interdisciplinary communication, inefficient documentation, and underuse of behavioral CPGs by nurses. In addition, the majority of the nurses agreed on 4 missing safety features of alerts and reminders within the system and the use of workarounds. Overall satisfaction with the system was just above average.

Our findings were consistent with common areas identified by usability studies of nursing documentation systems related to the long time for documentation and task completion, lack of data relevancy, and nurse perception of lack of system effect on quality of care [24,31,32,37,38]. Yet, our study was specific to the usability of a CPG-based care planning solution within the nursing documentation system and found a lack of system safety features as a major concern for nurses. The need for the KBC system to suggest critical interventions and alert nurses on safety considerations and inappropriate selection of CPGs was perceived as essential by almost all nurses.

Consistent with the findings from the rated survey items, common themes on negative aspects of the system in the narrative data were related to unnecessary repetitive documentation, difficulty finding appropriate CPGs, missing unit-specific information, irrelevancy of information, and the lack of perceived system value. These findings may explain the use of workarounds and inappropriate system use, such as using the system at the end of the shift for documentation purposes to cover nurses legally instead of using it to guide nursing care and the decision-making process. These forms of workarounds are examples of inappropriate use of EHR and are classified by Sittig and Singh [39,40] as EHR-related errors.

Although nurses recognized the system value on standardizing and individualizing care, 80.0% (80/100) did not believe that the quality of nursing care is based on system use. The difficulties nurses face in system use might mask the perceived effect of the system on improving patient outcomes and the value of nursing documentation on patient outcomes. The most commonly reported difficulty was repetitive documentation. Duplicate documentation is not only time consuming but also error-prone and, in our study, resulted from (1) lack of seamless data transmission and lack of communication across CPGs and flowsheets, (2) irrelevancy of CPGs to specific ICUs, which resulted in searching a long list to find an appropriate CPG and searching long lists of interventions and assessment, and (3) the need to go out of Sunrise to select CPGs. Another commonly cited downside of the system was lack of support to nursing information needs by missing important CPGs for some critical cases.

Although no significant correlations were found between years of experience and nurse satisfaction with the system, the value of the system to new nurses with less ICU experience and the lack of system value to expert nurses were supported by different comments from novice and expert nurses. This may support the difference in information need and the decision-making process between novice and expert ICU nurses. It may also suggest that expert ICU nurses appreciate systems that promote safety and efficient documentation, present only relevant information in a visible and easy-to-find manner, and allow nurses to have a sense of control in system use instead of searching long lists. Another possible explanation is the lack of appreciation among expert nurses that new evidence continues to change the way we provide care. One of the expert nurses commented, “You can perform without referring to KBC, if familiar with the interventions.”

Consistent with previous studies [37,38], our results supported the need for periodic training on system use. Almost 60% of the nurses reported they would benefit from refresher training sessions. Nurses’ inability to locate CPGs and the documentation of the multidisciplinary team reflects the difficulty in system use and supports the need for training. The reported difficulty in locating behavioral CPGs is a plausible explanation for behavioral CPG underuse. Another possible explanation is the complexity of medical conditions in ICUs that requires heavy reliance on medical-surgical CPGs for life-threatening conditions (eg, dysrhythmia). In the narrative data, one of the nurses commented on excessive documentation of 11 to 12 problems per patient. Managing and documenting the assessment, interventions, and patient education for that many problems is unrealistic in ICUs and suggests the need for training on system use. Nurses were educated to focus on 3 to 5 high-priority problems in patient care. On the contrary, nurses’ concerns about the legal aspects of documentation might explain the selection of multiple CPGs that would result in unmanageable care and excessive documentation. In addition, although the system is missing some CPGs for critical patient conditions often seen in ICUs, some of the CPGs reported by nurses as missing are actually available within the system, such as mechanical ventilation, neurological diseases, multiple sclerosis, and embolic stroke. This can be explained by the long list of CPGs and lack of automatic integration or suggestions of CPGs based on patient conditions and further supports the need for training on system use.

The use of a questionnaire in this study provided valuable input on system deficiencies and set the stage for future initiatives on observing user-system-task-environment interaction. This study provides valuable information for end users, leaders, researchers, stakeholders, and system vendors on strategies for system and workflow redesign improvement. The study identified usability issues that complicate nurses’ work, threaten appropriate system use, and initiate unsafe workarounds in complex ICU environments. In summary, usability issues identified by nurses in this study reflect system failure to achieve at least 10 of Zhang and Walji’s 14 usability principles [36] and suggest an urgent need for system redesign. For example, difficulties in finding CPGs negatively affect the *system* *visibility* principle. The moderate agreement to the statement “method and sequence of data entry match the workflow and thought processes of the nurse” and irrelevancy of data displayed by the system suggest a *mismatch between the system and nursing world*. Lack of safety features and workarounds are indicators of lack of *informative feedback*, inability to *prevent user errors*, and unavailability of *error message* principles. Nurses’ inability to discontinue one aspect of a CPG negates *flexibility and customizability* and *user control* principles. Inefficient documentation indicates system failure of the *help and documentation* principle. Lack of seamless data transition across CPGs and flowsheets and excessive data entry increase *memory load* and invalidate the *minimalist design* principle.

### Implications

Nursing documentation has safety, compliance, nursing and interdisciplinary communication, legal, accreditation, and financial implications for practitioners, administrators, researchers, and accreditation, safety, and reimbursement agencies. The Joint Commission requires the use of individualized plan of care for each patient to promote effective, continuous, and safe care. The use of EHR-integrated CPGs and CPG-based care planning IT solutions is essential to evidence-based and safe practice, individualized patient care, and complete and standardized documentation. However, inappropriate design, integration, and use of care planning systems such as the KBC would mask any relationships between CPG use and effective and complete documentation; CPG use and quality and safety of care; and complete documentation and safety and quality of care. To be effective, vendors and health care leaders should make certain that CPG-based care planning systems suggest critical interventions and alert nurses on safety considerations and inappropriate selection of CPGs; include a complete list of CPGs for ICU medical conditions; have a search engine for nurses to easily locate relevant unit-specific CPGs; and allow communication across CPGs to eliminate unnecessary repetitive documentation. IT and quality improvement departments and researchers are tasked to conduct periodic examination of nurses’ perceptions and use of the system, workarounds, as well as periodic training on system use as critical factors for system success.

### Limitations

The findings of this study should be interpreted in light of the following limitations. The study was implemented in ICUs where urgency of care, pressure to find relevant and supportive information, and efficiency of documentation are crucial. Nurse perception of the usability of the same system in other units with less critical care needs might be different. Although our data collection procedure was successful to achieve our target sample size, increasing the sample of ICU nurses and including non-ICU nurses may increase the generalizability of the study. The high response rate may also reflect nurse frustration with the system and the urgent need for system redesign. Finally, nursing care plan and documentation systems vary widely across health institutions in terms of technical complexity, customizability, amount, relevancy, visibility, organization, sources, credibility, and transition of information, safety features, and interoperability between nursing documentation systems and other modules in an EHR. This introduces a challenge for direct comparison across studies of different systems or even the same system with different implementation and EHR-integration frameworks. Nevertheless, the comparison can be made using usability principles.

### Conclusions

CPG-based care planning systems provide nurses access to easy-to-understand recommendations from hundreds of CPGs without the complexity of statistical jargons. Nevertheless, nurses’ perceptions of the usability of these systems are essential for appropriate and safe system use as well as safety and quality of care. Periodic training on system use is necessary. Training should not be limited to technical aspects of system use but should also highlight system value to nursing and patient care. Nursing care plan systems with CPGs in ICUs should be easy to navigate; promote safety; support efficient documentation; present relevant, unit-specific, and easy-to-find information; endorse interdisciplinary communication; and improve safety and quality of care.

## Appendix

Multimedia Appendix 1 Nurse perception of the usability of the knowledge-based charting system (N=100).

Multimedia Appendix 2 Thematic categories of advantages of knowledge-based charting system (N=88 nurses).

Multimedia Appendix 3 Thematic categories of negative aspects of knowledge-based charting system (N=90 nurses).

## Abbreviations

CPG: clinical practice guideline
EBP: evidence-based practice
EHR: electronic health record
ICU: intensive care unit
IT: information technology
KBC: Knowledge-Based Charting
MICU: medical intensive care unit
NeuroICU: neuro intensive care unit
STICU: surgical trauma intensive care unit
TCICU: transplant and cardiac intensive care unit
