# Supplementary material for: Nurses’ Perceptions of a Care Plan Information Technology Solution With Hundreds of Clinical Practice Guidelines in Adult Intensive Care Units: Survey Study
Source: JMIR Hum Factors. 2019 Feb 12;6(1):e11846. doi: 10.2196/11846 (PMC6390187; doi:10.2196/11846)
Supplement: Multimedia Appendix 1 [file humanfactors_v6i1e11846_app1.pdf]

Multimedia Appendix 1: Nurse perception of the usability of the knowledge-based charting (KBC) system (N= 100)

| # | Item | Agree n (%) | Neutral n (%) | Disagree n (%) |
|---|---|---|---|---|
| 1 | It would be essential if the system suggests the most critical interventions to be selected from the list | 90 (90) | 8 (8) | 2 (2) |
| 2 | It would be essential if the system alerts me to critical safety considerations | 86 (86) | 10 (10) | 4 (4) |
| 3 | I can discontinue an old CPG[a] and add a new one without any difficulties | 84 (84) | 8 (8) | 8 (8) |
| 4 | It would be very helpful to be able to view a summary of changes in patient care plan | 82 (82) | 14 (14) | 4 (4) |
| 5 | It would be essential if the system notifies me if I selected inappropriate CPGs[a] for my patient | 80 (80) | 8 (8) | 12 (12) |
| 6 | KBC[b] includes CPGs[a] for the majority of patients' diagnoses we see in my unit | 74 (74) | 11 (11) | 15 (15) |
| 7 | CPGs[a] help me provide care for medical cases that I am not familiar with | 72 (72) | 10 (10) | 18 (18) |
| 8 | Terms used to reflect patients' problems, goals and interventions are consistent across different CPGs[a] | 70 (70) | 22 (22) | 8 (8) |
| 9 | I am a proficient (skillful) user of KBC[b] | 69 (69) | 25 (25) | 6 (6) |
| 10 | It is easy to document care using KBC[b] | 66 (66) | 18 (18) | 16 (16) |
| 11 | CPGs' index maintains consistency in the way it presents CPGs[a]' names or titles | 64 (64) | 20 (20) | 16 (16) |
| 12 | KBC[b] is missing important CPGs[a] for some patients' diagnoses we often see in my unit | 63 (63) | 18 (18) | 19 (19) |
| 13 | CPGs[a] include recommendations to enhance patient engagement in the care plan | 63 (63) | 18 (18) | 19 (19) |
| 14 | CPG[a] helps individualize the patient care plan | 63 (63) | 21 (21) | 16 (16) |
| 15 | KBC[b] improves the quality of nursing | 63 (63) | 19 (19) | 18 (18) |

| | | | | |
|---|---|---|---|---|
| | documentation | | | |
| 16 | KBC[b] helps provide a comprehensive nursing care plan without missing important goals or interventions | 63 (63) | 19 (19) | 18 (18) |
| 17 | Help resources are available for KBC[b] use | 59 (59) | 21 (21) | 20 (20 |
| 18 | I use workarounds when I interact with KBC[b] | 59 (59) | 24 (24) | 17 (17) |
| 19 | The way KBC[b] is integrated into the electronic medical record is easy to navigate and interact with | 58 (58) | 26 (26) | 16 (16) |
| 20 | I received adequate training on KBC[b] use | 58 (58) | 24 (24) | 18 (18) |
| 21 | All information in KBC[b] flowsheets is relevant to nursing care | 57 (57) | 16 (16) | 27 (27) |
| 22 | I feel I can benefit from refresher classes on KBC[b] use | 56 (56) | 19 (19) | 25 (25) |
| 23 | The way KBC[b] is interfaced with the electronic medical record supports my information needs and workflow | 54 (54) | 23 (23) | 23 (23) |
| 24 | I can see the value of my documentation using KBC[b] system on patient outcomes | 54 (54) | 30 (30) | 16 (16) |
| 25 | When locating a CPG[a] from the index, it is easy to select that CPG[a] | 54 (54) | 27 (27) | 19 (19) |
| 26 | Overall, I am satisfied with the KBC[b] system | 54 (54) | 25 (25) | 21 (21) |
| 27 | The method and sequence of data entry match the workflow and thought processes of the nurse | 52 (52) | 28 (28) | 20 (20) |
| 28 | In general, KBC[b] is user friendly and easy to use | 52 (52) | 31 (31) | 17 (17) |
| 29 | I believe KBC[b] is more than just a documentation system and has important effects on improving patient outcomes and quality and safety of care | 49 (49) | 23 (23) | 28 (28) |
| 30 | It is easy to locate a specific medical surgical CPG[a] from the CPGs[a] index | 48 (48) | 23 (23) | 29 (29) |
| 31 | I can easily locate the documentation of the | 44 (44) | 24 (24) | 32 (32) |

| | | | | |
|---|---|---|---|---|
| | multidisciplinary team who uses KBC[b] | | | |
| 32 | Other disciplines can easily locate the nursing documentation based on KBC[b] | 40 (40) | 28 (28) | 32 (32) |
| 33 | It is easy to locate a specific behavioral CPG[a] from the CPGs[a] index | 37 (37) | 35 (35) | 28 (28) |
| 34 | There are shortcuts to help decrease the time of documentation using KBC[b] | 29 (29) | 23 (23) | 48 (48) |
| 35 | KBC improves documentation efficiency | 26 (26) | 29 (29) | 45 (45) |
| 36 | I often use behavioral CPGs[a] in my care plan | 21 (21) | 24 (24) | 55 (55) |
| 37 | The quality of my work is based on using KBC[b] | 20 (20) | 26 (26) | 54 (54) |

[a] CPG, clinical practice guidelines

[b] KBC, knowledge-based charting
