# Supplementary material for: Nurses’ Perceptions of a Care Plan Information Technology Solution With Hundreds of Clinical Practice Guidelines in Adult Intensive Care Units: Survey Study
Source: JMIR Hum Factors. 2019 Feb 12;6(1):e11846. doi: 10.2196/11846 (PMC6390187; doi:10.2196/11846)
Supplement: Multimedia Appendix 2 [file humanfactors_v6i1e11846_app2.pdf]

Multimedia Appendix 2: Thematic categories of advantages of knowledge-based charting system (N= 88 Nurses)

Category	Examples	Number of nurses, ICU
Serves as a reminder and a guide to provide complete care	• "it could remind us of things that we might have forgotten to look for or didn't know about in the assessment and interventions"<br>• "guides nursing care in the assessment, intervention, planning, evaluation, and documentation"	6, Neuro<br>14, Surgical<br>11, Medical<br>8, Transplant<br>(Total= 39)
Organizes care toward individualized patients' outcomes	• "organizes patient care"<br>• "helps individualize care, specific outcomes for each patient that need to be reached during inpatient stay"	3, Neuro<br>8, Surgical<br>5, Medical<br>8, Transplant<br>(Total= 24)
User friendly	• "user friendly"<br>• "easy to complete"	1, Neuro<br>2, Surgical<br>1, Medical<br>4, Transplant<br>(Total= 8)
Improves accountability	• "certain aspects of charting are covered in a legal standpoint"<br>• "keeps people accountable for patient care"	1, Neuro<br>1, Surgical<br>2, Medical<br>2, Transplant<br>(Total= 6)
Promotes evidence-based care	• "evidence-based and helps standardize care"<br>• "gets updated regularly for new treatment modalities"	4, Transplant<br>(Total= 4)

Educates nurses	• "allows newer nurses to focus on required interventions especially when working with a patient with diagnosis that they have never worked with before. It did that for me when I was a new nurse"<br>• "as a new nurse, the system helps me recall important assessments necessary to provide quality nursing care"<br>• "prompts some interventions to do with certain guidelines that I'm not familiar with"	2, Neuro<br>1, Surgical<br>(Total= 3)
