# Supplementary material for: Nurses’ Perceptions of a Care Plan Information Technology Solution With Hundreds of Clinical Practice Guidelines in Adult Intensive Care Units: Survey Study
Source: JMIR Hum Factors. 2019 Feb 12;6(1):e11846. doi: 10.2196/11846 (PMC6390187; doi:10.2196/11846)
Supplement: Multimedia Appendix 3 [file humanfactors_v6i1e11846_app3.pdf]

Multimedia Appendix 3: Thematic categories of negative aspects of knowledge-based charting system (N= 90 Nurses)

Category	Examples	Number of nurses, unit
Duplicate charting and time consuming	• "extremely repetitive, tedious and time consuming, especially when we have 11-12 problems per patient, wish the documentation slave over"<br>• "CPGs[a] do not communicate with each other. If they would somehow transfer, it would help nurses spend more time with patients and less time charting"	18, Neuro<br>20, Surgical<br>20, Medical<br>18, Transplant<br>(Total= 76)
Difficulty finding appropriate/specific CPGs	• "multiple diagnoses, no cross referencing- makes it very difficult to find CPGs[a]"<br>• "there are no descriptions to guide the nurse in selecting the correct CPG[a]"<br>• "searching a long list slows me down, this system needs search engine"	10, Neuro<br>16, Surgical<br>5, Medical<br>5, Transplant<br>(Total= 36)
Missing unit-specific CPGs	• "not all CPGs[a] are present and not all elements of the same CPG[a] exist"<br>• "missing different diseases and diagnoses"	10, Neuro<br>8, Medical<br>11, Transplant<br>(Total= 29)
Too broad and irrelevant	• "irrelevant and not unit-specific"<br>• "several items are not applicable to critical care, such as nutrition goals. Many patients are not eating or only tube feeds"<br>• "have to scroll down the entire list to add a simple intervention like a turn or blood draw"	12, Neuro<br>7, Surgical<br>4, Medical<br>5, Transplant<br>(Total= 28)
Has no value to patient care or	• "does not change or improve patient outcomes"<br>• "we feel like its just for the Joint Commission"	4, Neuro<br>7, Surgical

nursing	• “it is just used for legal purposes in case I go to court”<br>• “sometimes I seem to settle on a CPG[a] just to have one; feel like the CPGs[a] are never part of changing the plan of care”<br>• “I have never viewed it to improve safety or quality, just another hoop to jump through and another thing to chart with no purpose”<br>• “I don't feel it really helps improve patients' safety or quality of care because we don't usually have time to look at it until the end of our shift when we are having to fill it out”.<br>• “I use protocols than anything; I very rarely look at KBC[b] or CPGs[a], only when I need to chart on it”<br>• “not every nurse completes the documentation”<br>• “if familiar with interventions, can perform without referring to KBC[b]”<br>• “does not have much value for experienced nurse”<br>• “it is a formality for how we should document more than a guide”	8, Medical<br>6, Transplant<br>(Total= 25)
Not user friendly	• “over complicated and difficult to navigate”<br>• “have to click out of Sunrise to select CPGs[a]”<br>• “you can’t discontinue one aspect of it”	3, Neuro<br>6, Surgical<br>8, Medical<br>3, Transplant<br>(Total= 20)
Lacks safety features	• “it does not prompt you when there is something not done”<br>• “lacks warnings or alerts for critical interventions”	1, Surgical<br>2, Transplant<br>(Total= 3)

No sufficient training	• "I'm a new nurse and there hasn't been enough training regarding use"<br>• "did not get training on behavioral responses CPGs[a]"	I, Neuro<br>I, Surgical<br>1, Medical<br>(Total= 3)

[a] CPG, clinical practice guidelines

[b] KBC, knowledge based charting
